# C-Terminal Redox Domain of *Arabidopsis* APR1 Is a Non-Canonical Thioredoxin Domain with Glutaredoxin Function

**DOI:** 10.3390/antiox8100461

**Published:** 2019-10-08

**Authors:** Fang-Fang Chen, Chia-Yu Chien, Chao-Cheng Cho, Yu-Yung Chang, Chun-Hua Hsu

**Affiliations:** 1 Department of Agricultural Chemistry, National Taiwan University, Taipei 10617, Taiwan, fun_0108@hotmail.com (F.-F.C.); chiayu31.cc@gmail.com (C.-Y.C.); yuyungchang@gmail.com (Y.-Y.C.); 2 Genome and Systems Biology Degree Program, National Taiwan University and Academia Sinica, Taipei 10617, Taiwan; chaochengcho@gmail.com

**Keywords:** APS reductase, redox domain, crystal structure, sulfur assimilation, redox potential

## Abstract

Sulfur is an essential nutrient that can be converted into utilizable metabolic forms to produce sulfur-containing metabolites in plant. Adenosine 5′-phosphosulfate (APS) reductase (APR) plays a vital role in catalyzing the reduction of activated sulfate to sulfite, which requires glutathione. Previous studies have shown that the C-terminal domain of APR acts as a glutathione-dependent reductase. The crystal structure of the C-terminal redox domain of *Arabidopsis* APR1 (AtAPR1) shows a conserved α/β thioredoxin fold, but not a glutaredoxin fold. Further biochemical studies of the redox domain from AtAPR1 provided evidence to support the structural observation. Collectively, our results provide structural and biochemical information to explain how the thioredoxin fold exerts the glutaredoxin function in APR.

## 1. Introduction

Sulfur, which is a crucial element in life, can be incorporated into the amino acids cysteine and methionine, iron-sulfur clusters, and other cofactors [1,2]. In fact, a variety of S-containing secondary metabolites are produced, which usually play a significant role in defense against pathogens and herbivores in plant [3,4]. For incorporation into bioorganic compounds, oxidized inorganic sulfur compounds (usually sulfate) need to be reduced in the pathway of assimilatory sulfate reduction.

Sulfate is first activated by adenosine triphosphate (ATP) sulfurylase, forming 5′-adenylylsulfate (APS), which alternatively can be phosphorylated by APS kinase and forms 3′-phosphoadenosine-5′-phosphosulfate (PAPS). Generally, it was accepted that plants, algae, and phototrophic bacteria use APS for assimilatory sulfate reduction, whereas bacteria and fungi use PAPS. A two-step catalytic process was first demonstrated in *Arabidopsis*, then in a prokaryotic organism, *Mycobacteria tuberculosis*. The first step is the reaction between the key enzyme APS reductase (APR) and APS to generate a thiosulfonate-enzyme intermediate. Next, in prokaryotes, the release of sulfite from the intermediate complex is mediated by thioredoxin [5]. In plants, the C-terminal domain of APR itself serves the function like thioredoxin. The importance of the C-terminal domain was verified by heterologous fusion of the plant APR C-terminal domain to bacterial APR. The recombination resulted in the thioredoxin-dependent enzyme having glutathione (GSH)-utilizing ability [6].

**Figure 1:**
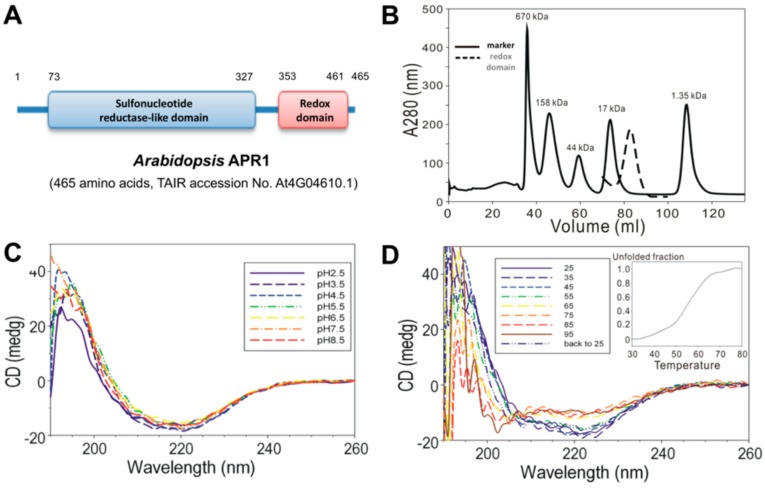
Analysis of biochemical properties of *Arabidopsis thaliana* APR1 (AtAPR1) C-terminal redox domain. (**A**) Schematic diagram of the domain organization of *Arabidopsis* APR1. (**B**) Size-exclusion chromatography results of AtAPR1 redox domain (black line) and markers (gray dots). The standard markers (BioRad) are bovine thyroglobulin (670 kDa), bovine gamma globulin (158 kDa), chicken ovalbumin (44 kDa), horse myoglobin (17 kDa), and vitamin B_12_ (1.35 kDa). The peak elution at 82.86 mL is the predicted size for a monomer of the redox domain with estimated molecular mass 13 kDa. (**C**) Folding of the AtAPR1 redox domain measured by circular dichroism (CD) spectroscopy. The CD spectra for the AtAPR1 redox domain show a stable fold at various pH values. (**D**) The CD spectra for thermal denaturation of the AtAPR1 redox domain presents a highly thermos-reversible property. Inset: melting temperature can be roughly estimated as 55 °C.

Plant-type APR is necessary for plant growth [7] and tolerance to heavy metals [8], and directly connects the sulfur assimilation pathway [7,9,10,11,12]. Physiologically, APR is upregulated under sulfur-deficient conditions and downregulated on exposure to organosulfur compounds such as GSH or under CO_2_ and nitrogen insufficiency [10,13,14]. This key enzyme in sulfur assimilation is also tightly regulated by other environmental stimuli such as salinity, plant hormones, and light [10,14]. The overexpression of APR would cause metabolic changes leading to the accumulation of reduced sulfur metabolite activity, resulting in damaged plant tissue and decreased plant growth [15,16]. Hence, APR activity is downregulated to avoid tissue injury by a negative feedback regulation, a mechanism associated with metabolic changes that cause reduced GSH concentration and the accumulation of sulfate [8,17]. In contrast to the cooperation of a sulfonucleotide reductase and a thioredoxin in prokaryote systems, in plants, the protein involved in the pathway is only a single polypeptide which consists of two distinct domains: a sulfonucleotide reductase-like one and a thioredoxin-like one (Figure 1A). A C-terminal truncated plant-type APR lacking the redox domain losses APR activity but recovers the function after the addition of thioredoxin [18,19].

Sequence alignment revealed that the C-terminal redox domain of APR is more similar to thioredoxin than glutaredoxin. However, it possesses enzymatic activity similar to glutaredoxin [19,20]. Mechanically, glutaredoxin does not require enzymatic reduction like thioredoxin by thioredoxin reductase, but its activity is reduced by a redox cofactor, GSH. To gain more insights into the C-terminal redox domain of plant-type APR, here we present a comprehensive structural and biochemical study of *Arabidopsis thaliana* APR1 (AtAPR1). To obtain further detailed information, the x-ray crystal structure of the APR1 redox domain was determined. The evidence supporting a glutaredoxin function for the redox domain was confirmed by isothermal titration calorimetry (ITC) and redox potential assay. Furthermore, the comparison of the AtAPR1 redox domain to other structural homologues helped us clarify that the AtAPR1 redox domain with protein sequence similarity to thioredoxin possesses glutaredoxin activity.

## 2. Materials and Methods

### 2.1. Protein Expression and Purification

The DNA sequence containing the C-terminal redox domain (amino acids 353–461) of AtAPR1 (TAIR accession no.: At4G04610.1) was amplified by PCR. The redox domain fragment was inserted between the *Nde*I and *Xho*I sites of the pGEX-4T1 vector system (GE Healthcare, Marlborough, MA, USA) as described [21]. The forward and reverse PCR primers used for amplification were APR1C-F (5′-AATTGAATTCATGGAGAATCTTGTGACTTTG-3′) and APR1C-R (5′-AATTCTCGAGCAAGAACGAAGTCAAAGACT-3′). Escherichia coli BL21(DE3) cells transformed the resulting plasmid were grown at 37 °C up to OD_600_ 0.6 with 50 μg/mL ampicillin. The expression of the recombinant AtAPR1 redox domain with an GST-tag at the N-terminus was induced in cells with 1 mM isopropyl-β-D-thiogalactoside, followed by growth for 17 h at 25 °C. Cells were collected by centrifugation and resuspended in lysis buffer (25 mM phosphate buffer, pH 7.4, 140 mM NaCl). The cells were disrupted by sonication and then centrifuged to remove debris. The clear supernatant was loaded into a column filled with GSH-Sepharose resin (GE Healthcare). The resin was washed with a 10-times volume of lysis buffer and the GST-tagged AtAPR1 redox domain was eluted by elution buffer (50 mM Tris-HCl pH 8.0, 10 mM GSH). The GST-tag was removed by using thrombin, which artificially resulted in six additional residues (GSPEFM) at the N terminus.

### 2.2. Size Exclusion Chromatography

The resulting redox domain protein was further purified and size-determined by gel filtration chromatography with a HiPrep 16/60 Sephacryl S-200 HR column (GE Healthcare) connected to the AKTA FPLC system (GE Healthcare, MA, USA) in gel filtration buffer (20 mM Tris-HCl buffer, pH 7.0, 200 mM NaCl). The elution profile of the AtAPR1 redox domain was determined to estimate molecular weight by comparison with the elution volumes of standard proteins (biorad 151-1901), including bovine thyroglobulin (670 kDa), bovine gamma globulin (158 kDa), chicken ovalbumin (44 kDa), horse myoglobin (17 kDa), and vitamin B_12_ (1.35 kDa). The collected fraction was concentrated to 3.5 mg mL^−1^ and determined by the Bradford method [22]. The purity of the AtAPR1 redox domain was estimated to be 98% by Coomassie blue-stained SDS-PAGE analysis.

### 2.3. Circular Dichroism (CD) Spectroscopy

Far-UV CD spectra of 20 μM protein samples in various pH value were measured and recorded on a JASCO J-810 spectropolarimeter equipped with a Peltier temperature control system (JASCO International Co., Tokyo, Japan). All CD data were converted from CD signal (millidegree) to mean residue ellipticity (deg⋅cm^2^⋅dmol^−1^) after applying background subtraction. The CD spectra were analyzed for content of protein secondary structure by using CDPro software [23]. Thermal transition of protein samples was calculated by the CD spectra recorded every 10 °C from 25 °C to 95 °C.

### 2.4. Crystallization, Crystal Data Collection, Structure Determination, and Refinement

With use of a HoneyBee 963 robot (Genomic Solutions), initial protein crystallization trials were performed at 283 K by using the sitting-drop vapor-diffusion method. The crystals for data collection were grown at 283 K in one week with the optimal condition of 100 mM Tris buffer, pH 7.0, 1.0 M sodium citrate, and 200 mM sodium chloride. The crystal was cryoprotected in mother liquor supplemented with 20% glycerol and flash-frozen in liquid nitrogen at 100 K. The data from diffraction images were collected at beamline BL13B1 (National Synchrotron Radiation Research Center, Taiwan) and processed by using HKL2000 software [24]. The crystal structure of the AtAPR1 redox domain was solved by the molecular replacement method by using BALBES [25]. Six structures with sequence identity >20% were found. The best solution involved using the J-Trx1 fragment of protein disulphide reductase ERdj5 from *Mus musculus* (PDB entry 3APQ; sequence identity 25%) [26]. The models were refined by using the phenix.refine program [27] in the PHENIX package [28] interspersed with manual inspection and corrections by using COOT [29]. During the later stages, restrained positional and B-factor refinement involved using the program phenix.refine. The models were evaluated with the use of MOLPROBITY [30] and PROCHECK [31] (Supplementary Figure S1). The data collection and structure refinement statistics are in Table 1. Atomic coordinates and crystallographic structure factors have been deposited in the PDB under the accession code 5YRY. 

### 2.5. Isothermal Titration Calorimetry (ITC)

Binding of GSH or GSSG to the AtAPR1 redox domain was measured by using ITC with the Nano ITC system (TA Instruments). Aliquots of 5 μL of 1 mM ligand were titrated by injection into protein (0.03 mM in 1.3 mL buffer containing 20 mM Tris-HCl, pH 8.0, 100 mM NaCl). Experiments were conducted with 250 rpm stirring at 25 °C. Finally, the corrected heat from the binding reaction with background heat subtraction was used to derive values for the stoichiometry of the binding (*n*), dissociation constant (*Kd*), apparent enthalpy of binding (*ΔH*), and entropy change (*ΔS*). Data were fitted by applying a site-specific binding model with NanoAnalyze v2.4.1 software.

### 2.6. Redox Potential Assay

The in vitro redox state of the recombinant AtAPR1 redox domain was measured as described [32]. In this assay, AtAPR1 redox domain protein (1 μM) was incubated with 0.1 mM GSSG at 25 °C and varying concentrations of GSH in 100 mM PBS (phosphate buffered saline, pH 7.0) containing 1 mM EDTA for 1 h. To stop the reaction, 10% trichloroacetic acid was added to prevent further thiol-disulfide exchange. After centrifugation and removing the supernatant, the precipitated pellet was solubilized in 100 mM PBS containing 2% SDS and 1 mM AMS (4-acetamido-4′-maleidylstilbene-2,2′-disulfonic acid), then incubated at 25 °C for 1 h to alkylate free sulfhydryl groups of cysteine. For visualization, the samples were separated by 12.5% SDS-PAGE and stained with Coomassie Brilliant Blue. The ratio of reduced form was compared with the intensities of bands on the gel and quantified by using ImageJ [33]. The redox equilibrium constant (*K*_eq_) was calculated by fitting the fraction of the reduced form to the following equation: *r* = ([GSH]^2^/[GSSG])/(*K*_eq_ + ([GSH]^2^/[GSSG])) [34], where *r* is the relative ratio of reduced AtAPR1 redox domain. The GSH/GSSG redox potential was further analyzed according to the Nernst equation (*E_h_* = *E_0_* + 2.3 × *RT*/*Nf* × log([GSSG]/[GSH]^2^), where *E_0_* = –240 mV at pH 7.0 [34] and *n* =2 for the two-electron oxidation of 2GSH to GSSG.

### 2.7. Molecular Modeling of Protein Complex 

GSH was docked into the AtAPR1 redox domain active site by manual docking, superimposing the structures of glutaredoxin bound with GSH on that of the AtAPR1 redox domain. The structure of cGrx1 complexed with GSH (PDB code 4TR1) [35] was used as a template. Further energy minimization involved using GROMACS [36]. The interaction between GSH and the AtAPR1 redox domain was analyzed by using Ligplot+ [37]. 

## 3. Results and Discussion

### 3.1. Biochemical Properties of AtAPR1 C-Terminal Redox Domain

The DNA encoding amino acids 353 to 461 (C-terminal redox domain) of *Arabidopsis thaliana* APR1 were cloned into a bacterial expression plasmid for protein production. With the N-terminal GST-tag, the recombinant AtAPR1 redox domain could be purified by using a GST-affinity column. The GST-tag was then excised from the AtAPR1 redox domain with thrombin protease, which resulted in a Gly-Ser-Pro-Glu-Phe-Met extra sequence at the N-terminus. Gel filtration chromatography was used to isolate a homogenous protein sample. The molecular weight of the recombinant AtAPR1 redox domain was calculated from the amino acid sequence (13,046 Da) and considered similar to the estimation as 13 kDa by SDS-PAGE. The molecular mass of the AtAPR1 redox domain in solution determined by size-exclusion chromatography was about 13 kDa (Figure 1B), which indicates the monomeric form of the AtAPR1 redox domain. Because plant APR1 is arranged as oligomers [38,39], this result implied that the oligomerization of AtAPR1 is not formed via redox domain self-interaction.

By using far-UV CD spectroscopy, we measured the extent of secondary structural changes in the AtAPR1 redox domain at different pH values and temperatures. The redox domain showed a typical α/β type signal in a far-UV CD spectrum (Figure 1C, red dashed line). Additionally, the far-UV CD spectra for the redox domain were almost indistinguishable (identical) at various pH values, showing one maximum at 195 nm and two minima, at 208 and 220 nm (Figure 1C). These results suggest that the overall secondary structure of the protein was not disrupted with lowering the pH value. Furthermore, the contents of the α-helix and β-strand of the redox domain estimated by CDPro software [23] are 36% and 20%, respectively. The secondary structure of the redox domain was greatly distorted on heating to 55 °C by estimating from a series of CD spectra at various temperatures (Figure 1D, inset). Remarkably, the CD spectra for the AtAPR1 redox domain, which was 95 °C thermal-denatured followed by cooling to 25 °C, was almost identical to that of the native AtAPR1 redox domain measured at 25 °C. This indicates that thermal denaturation of the redox domain is reversible. 

### 3.2. Overall Structure of AtAPR1 Redox Domain

To provide insights into the structural basis of the AtAPR1 redox domain, we produced and crystallized the C-terminal redox domain of *Arabidopsis* APR1 (residues 353–461) [21] and obtained crystal belonging to the tetragonal space group with the following unit-cell dimensions: *a* = *b* = 58.2, *c* = 86.7 Å. Molecular replacement was applied to determine the phase by using the BALBES program [25]. For template searching, six structures with sequence identity > 20% were found. The best solution was found by using the J-Trx1 fragment of protein disulphide reductase ERdj5 from *Mus musculus* (PDB code, 3APQ; sequence identity 25%) [26]. This solution gave a monomer in the space group *P*4_3_2_1_2, with a final R factor and *R_free_* of 36.9% and 45.9%, respectively, and a Q factor of 0.666 after simple refinement by REFMAC5 [40]. Phaser [41] was used to fit the PDB model output from BALBES into the tetragonal 2.70-Å resolution data, with a TFZ score of 12.7 and an LLG of 731. Finally, the crystal structure of the AtAPR1 redox domain was refined to *R_work_* = 17.9% and *R_free_* = 25.0% with high-quality backbone geometry checked by Ramachandran plot (Figure S1). A summary of data collection and refinement statistics is given in Table 1.

**Figure 2:**
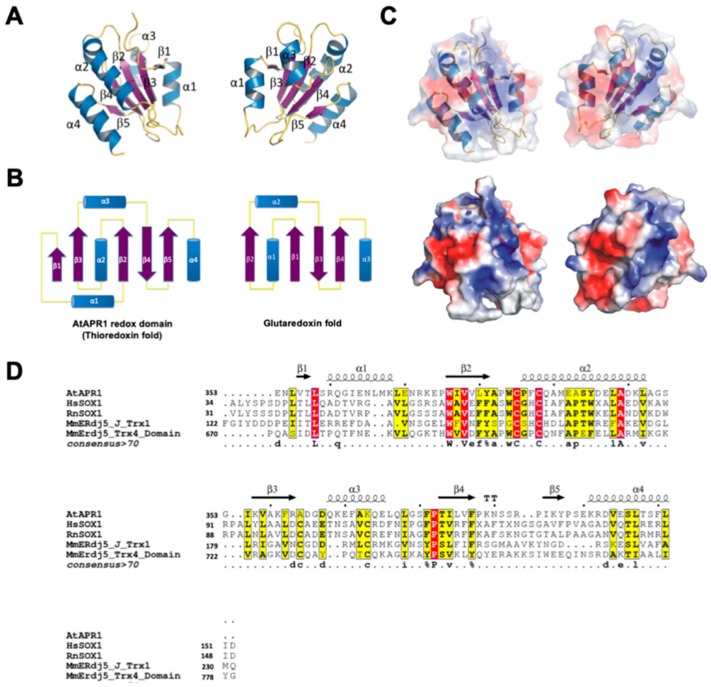
Crystal structure of the C-terminal redox domain of AtAPR1. (**A**) Structure of the AtAPR1 redox domain displays a typical thioredoxin fold with a central five-stranded β-sheet (β1–β5) and four flanking α-helices (α1–α4). Helices, strands, and loops are shown in blue, purple, and yellow, respectively. (**B**) Topology diagram of the AtAPR1 redox domain and glutaredoxin fold. (**C**) The electrostatic surface potentials mapped with red patches and blue patches represented as negative and positive charge, respectively. (**D**) Structure-based sequence alignment of the AtAPR1 redox domain with other structural homologs. Selected proteins shown in alignment are human sulfhydryl oxidase 1 (hsSOX1; PDB code: 3Q6O), rat sulfhydryl oxidase 1 (RnSOX1; PDB code: 4P2L), mouse ER-resident protein disulfide reductase (MmERdj5) J-Trx1 fragment (PDB code: 3APQ), and mouse ER-resident protein disulfide reductase (MmERdj5) TRX4 domain (PDB code: 3APS). Identical residues are in white and the frame is in red. Amino acids with similarity score > 0.7 are framed in yellow. Secondary structure of the AtAPR1 redox domain is drawn above the alignment with arrows as sheets and strings as helices.

The structure of the AtAPR1 redox domain is a compact spherical molecule comprising a central core of five-stranded β-sheets flanked on either side by four helices (Figure 2A). The fold of the redox domain arranged in the order β1-α1-β2-α2-β3-α3-β4-β5-α4 is similar to the thioredoxin fold but not glutaredoxin fold (Figure 2B). The N-terminal region begins with a short β1 strand (residues Val4-Leu6), followed by α1 and β2, consisting of residues Arg8 to Lys16 and Trp24 to Tyr29, respectively. The redox-active motif (Cys33-Pro34-Phe35-Cys36) is located at the N-terminal end of the α2-helix, consisting of residues Pro34 to Leu50. The strand β3 comprises residues Lys56 to Arg61, followed by β4 (Thr81-Phe85), β5 (Ile93-Tyr95) and a C-terminal helix which consists of residues Lys99 to Glu111. Strands β1, β2 and β3 are parallel, and strand β4 is antiparallel to β2 and β5. Helices α1 and α3 pack on one side of the central β-sheet, whereas helices α2 and α4 are located at opposite sides. The packing of the sandwich-like architecture is mainly maintained by hydrophobic interactions between the sheet and helices (Figure 2A). Surface potential distribution of the redox domain shows most positive-charged residues around the redox-active motif (Figure 2C). From the structural analysis, the AtAPR1 redox domain exhibits a completely different secondary structure composition from that of orthodox glutaredoxins (Figure 2B).

### 3.3. Structural Comparison of AtAPR1 Redox Domain

A search of the DALI database [42] with the structure of AtAPR1 redox domain used as a model revealed several structural homologs, although the Z-score and sequence identity were not high (all Z scores < 14% and sequence identities ≤ 25%) (Table S1). The top-ranked structures were thioredoxin domains of sulfhydryl oxidase or disulfide reductase such as those for human sulfhydryl oxidase 1 (PDB code, 3Q6O; Z-score 13.9; root-mean-square deviation [RMSD] 2.3; sequence identity 19%; sequence similarity 40%) [43], rat sulfhydryl oxidase 1 (PDB code, 4P2L; Z-score 13.8; RMSD 2.4; sequence identity 20%; sequence similarity 37%) [44], and mouse ERdj5 (ER-resident protein disulfide reductase) J-Trx1 fragment (PDB code, 3APQ; Z-score 13.7; RMSD 2.6; sequence identity 20%; sequence similarity 42%) [26], mouse ERdj5 TRX4 domain (PDB code, 3APS; Z-score 13.2; RMSD 2.6; sequence identity 20%; sequence similarity 45%) [26]. Other structural relatives on the DALI server were all thioredoxin-like domains (Supplementary Table S1). This finding indicates that the C-terminal redox domain of AtAPR1 is structurally well conserved with thioredoxin even though the protein sequence similarities are low between the AtAPR1 redox domain and thioredoxins (Figure 2D).

### 3.4. GSH Binding Ability of AtAPR1 C-Terminal Redox Domain

**Figure 3:**
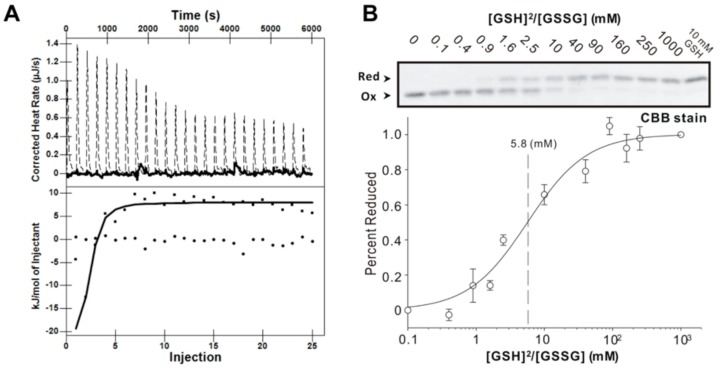
AtAPR1 redox domain possesses glutaredoxin activity. (**A**) Isothermal titration calorimetric thermogram (top) and isotherm (bottom) for the glutathioylation of the AtAPR1 redox domain. The dotted line in the thermogram is the adjusted calorimetric baseline. The solid line in the isotherm was obtained from nonlinear least-squares regression to both the OneSites and the Exchange model (the regression lines from the two algorithms coincide). (**B**) Determination of the equilibrium constant of the AtAPR1 redox domain with glutathione at pH 7.0 and 25 °C. (top) After incubation with different [GSH]^2^/[GSSG] ratios, the free sulfhydryl groups of the cysteine residues were modified with use of AMS (Bottom). A fraction of the reduced AtAPR1 redox domain was used to measure the redox equilibrium constant of the AtAPR1 redox domain. The apparent equilibrium constant between the AtAPR1 redox domain and glutathione was calculated by nonlinear least-squares fitting of the data in the upper column.

ITC measurements were used to further examine the equilibrium dissociation constant (Kd) of GSH and GSSG. A representative thermogram and the corresponding isotherm for the titration of 0.03 mM of the AtAPR1 redox domain with 1 mM GSH or GSSG obtained at 25 °C are shown in Figure 3A. ITC data indicated that GSH but not GSSG bound to the AtAPR1 redox domain with some enthalpy change (exothermic, *∆H* = –9.45 ± 2.17 KJ/mol). After fitting the ITC data, the *Kd* was determined to be 2.84 ± 0.29 µM (Table 2). The binding reaction at 25 °C was spontaneous with exergonic Gibbs energy of binding (*∆G* = -31.56 ± 0.13 KJ/mol). In addition, the thermodynamic profile (*∆G* < 0, *∆H* < 0, and –*T∆S* < 0) of GSH binding to the AtAPR1 redox domain suggests that GSH is likely stabilized by hydrophobic interaction [45]. The reaction is entropy driven with a large favorable entropy penalty (–*TΔS*= –23.92 ± 6.15 KJ/mol). This strongly favorable contribution from entropy to the glutathionylation reaction probably stems from conformational restrictions on the protein structure. 

### 3.5. Redox Potential of AtAPR1 Redox Domain

The redox equilibrium constants (*K*_eq_) of the AtAPR1 redox domain with GSH were calculated at pH 7.0 by measuring the formation of alkylated protein with reduction at a range of ratios of [GSH]^2^/[GSSG] [34]. The equilibrium measurement and the deduced redox potential of AtAPR1 redox domain are shown in Figure 3B and Table 3. By using the Nernst equation, the redox potential of –188 mV was further calculated. Intriguingly, the calculated redox potential of the AtAPR1 redox domain is more positive than that of plant thioredoxins (highest –368 mV) and compares well with that of *Saccharomyces cerevisiae* glutaredoxin (estimated at about –175 mV) (Table 3). This indicates that the cellular function of AtAPR1 redox domain may be similar to that of glutaredoxin. The AtAPR1 redox domain also exhibits a low redox *K*_eq_ of 5.8 mM, which indicates more oxidizing forms of protein under the physiological state because the normal GSH concentration in chloroplast is about 3 to 5 mM [46]. The properties of high redox potential and low redox *K_eq_* indicates that the AtAPR1 redox domain has a high tendency to be reduced by the cellular reductant GSH. This situation may enable the AtAPR1 redox domain to efficiently facilitate the thiol-disulfide exchange event under situations such as intracellular activation by oxidative stress and light responses in the photosynthetic apparatus, where GSH is largely accumulated [47].

### 3.6. Structural Model of GSH Bound to AtAPR1 Redox Domain

**Figure 4:**
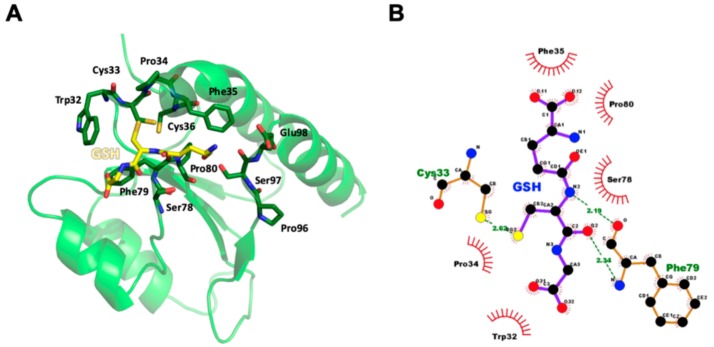
Molecular model of AtAPR1 redox domain in complex with GSH. (**A**) The cysteinyl moiety of GSH tri-peptide lies in a shallow groove formed by the Trp32-Cys33-Pro34-Phe35-Cys36, Ser78-Phe79-Pro80, and Pro96-Ser97-Glu98 loops of the redox domain. Hydrogen bonds are represented as dashed lines in black. (**B**) Interactions between the AtAPR1 redox domain and GSH were generated by using LigPlot+ [56]. GSH and nearby residues are represented as a ball-and-stick model with carbon in black, nitrogen in blue, oxygen in red, and sulfur in yellow. Interactions of GSH binding to the AtAPR1 redox domain via hydrogen bonds are shown in green with dashed lines and bond length as numeric numbers. Critical residues that offer hydrophobic interactions with GSH are in black with red eyelash symbols.

To elucidate the binding mode of GSH for the thioredoxin-like redox domain of AtAPR1, molecular docking was used to construct the complex model. The cysteinyl moiety of GSH tri-peptide lies in a shallow groove formed by the Trp32-Cys33-Pro34-Phe35-Cys36, Ser78-Phe79-Pro80, and Pro96-Ser97-Glu98 loops of the redox domain (Figure 4A). Several hydrogen bonds and hydrophobic interactions participated in GSH coordinating with the redox domain (Figure 4B). The sulfur atom in the cysteinyl moiety of GSH contacts the side chain of Cys33 via a hydrogen bond. The conserved proline at position 80 is a cis-configuration, which facilitates the backbone to assume the correct conformation for forming two hydrogen bonds between the main chain of Phe79 and the cysteinyl moiety of GSH. A short antiparallel β-sheet is packed between GSH and the cis-Pro loop, which is observed in all glutaredoxins and has been preserved or converged in evolution to form part of a substrate-binding site [55].

**Figure 5:**
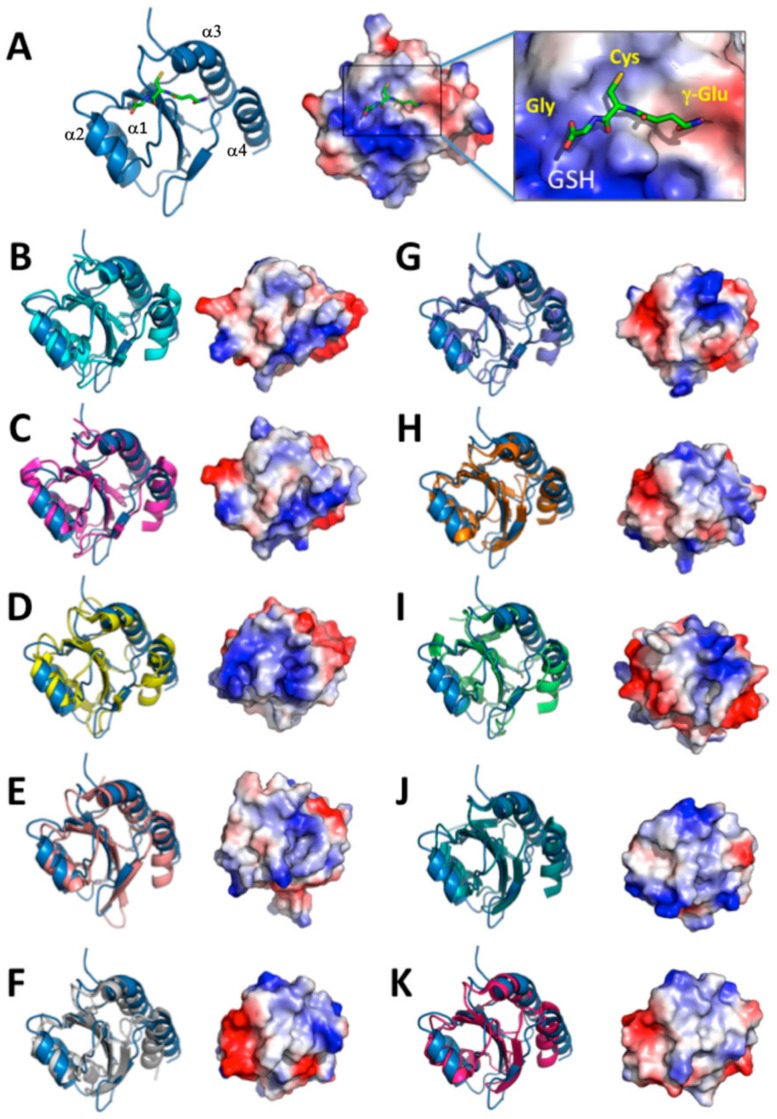
Comparison of structure and surface electrostatic potential of AtAPR1 redox domain with structural relatives. Electrostatic potentials (positive in blue and negative in red) of each molecule are mapped on the van der Waals surfaces. The PDB codes of the structural relatives are as follows: AtAPR1 redox domain (PDB code: 5YRY) (**A**), 3Q6O (**B**), 4P2L (**C**), 3APQ (**D**), 3APS (**E**), 1EP7 (**F**), 4EF0 (**G**), 2YOI (**H**), 2B5E (**I**), 2PPT (**J**), and 3M9J (**K**).

We chose the structures from the top 10 ranking results from a search of the DALI database and compared the structures with the AtAPR1 redox domain (Figure 5). Of note, variability between the AtAPR1 redox domain and all thioredoxin-like structures arises from the loops connecting the core secondary structure elements and results in different orientations of helices (α1, α3, and α4) (Supplementary Table S2). From this viewpoint, the AtAPR1 thioredoxin-like redox domain seems to adopt a non-canonical thioredoxin fold. Notably, the surface charge distribution around the GSH binding site of the AtAPR1 redox domain is ideally complementary to the charge of GSH, but other thioredoxins are not. This finding may explain why the GSH binder is the AtAPR1 redox domain instead of thioredoxins.

## 4. Conclusions

Although previous reports have confirmed that the carboxyl terminus of AtAPR1 efficiently utilizes GSH for sulfate reduction, the molecular details of how the thioredoxin structural homolog functions as a glutaredoxin were lacking. Here, we aimed to answer the questions with structural and biochemical approaches. As a whole, the structural information indicates that the C-terminal redox domain of plant-type APR1 adopts a non-canonical thioredoxin fold with exceptional orientations of helices α2, α3, and α4, which is quite different from typical thioredoxins. Further analysis of the surface charge distribution of the substrate binding site implies that GSH finely binds to the thioredoxin-like redox domain of AtAPR1 instead of other thioredoxins. Additionally, our biochemical studies suggest that the thioredoxin-like redox domain possesses glutaredoxin activity in vitro. In summary, our findings provide structural insights into the AtAPR1 redox domain and also significantly broaden our understanding of the activity of thioredoxin.

## Figures and Tables

**Table 1:** Data collection and refinement statistics of AtAPR1 redox domain.

Crystal Parameters	
Crystal	AtAPR1 redox domain
Space Group	*P*4_3_2_1_2
Unit cell parameters (Å)	*a* = *b* = 58.2; *c*= 86.7
Monomers per asymmetric unit cell	1
Data collection	
Wavelength (Å)	1.00
Resolution range (Å)	24.2–2.70 (2.80–2.70)
Unique no. of reflections	4467 (432)
Total no. of reflections	57471 (5450)
I/σ ^a^	32.4 (4.7)
*R*_merge_^a,b^	0.079 (0.660)
Completeness (%)	99.6 (100.0)
Refinement statistics	
Resolution (Å)	2.70
*R*_work_ (%)/*R*_free_ (%) ^c^	17.93/25.04
RMSD	
Bonds (Å)	0.008
Angles (^o^)	1.19
Mean B-factor (Å^2^)	72.0
Protein	72.0
Water	66.7
Ramachandran plot (%)	
Favored	97.0
Allowed	3.0
Outliers	0.0

^a^ Values in parentheses are for the highest resolution shell; ^b^ *R*_merge_=Σ_h_Σ_i_|*I_h_,_i_-I_h_*|/Σ_h_Σ_i_*I_h_*,*_i_*, where *I_h_* is the mean intensity of the *i* observations of symmetry-related reflections of *h*; ^c^ *R*_work_/*R*_free_=Σ|*F_obs_*-*F_calc_*|/Σ*F_obs_*, where *F_calc_* is the calculated protein structure factor from the atomic model (*R*_free_ was calculated with 5% of the reflections selected); RMSD, root-mean-square deviation.

**Table 2:** Thermodynamic parameters for binding of AtAPR1 redox domain to GSH or GSSG.

Ligand	*Kd* (µM)	*n*	*∆H* (KJ/mol)	–*T∆S* (KJ/mol)	*∆G* (KJ/mol)
GSH	2.84 ± 0.29	0.90 ± 0.03	−9.45 ± 2.17	−23.92 ± 6.15	−31.56 ± 0.13
GSSG	ND	ND	ND	ND	ND

All experiments were performed at 25 °C. Values are mean ± SD of three independent experiments. ND, not detected.

**Table 3:** Midpoint redox potentials of AtAPR1 redox domain and several reported thioredoxins and glutaredoxins.

Protein	Organism	Localization/type	Midpoint Redox Potentials (E_m,7.0,_ mV)	References
Trx-*m2*	*A. thaliana*	Chloroplastic thioredoxin	−368	[48]
Trx-*f1*	*A. thaliana*	Chloroplastic thioredoxin	−351	[48]
Trx-*m*	*S. oleracea*	Chloroplastic thioredoxin	−300	[49]
AtTDX	*A. thaliana*	Cytosolic and nuclear thioredoxin	−260	[50]
Trx-*z*	*P. trichocarpa*	Chloroplastic thioredoxin	−251	[51]
AtACHT4	*A. thaliana*	Chloroplastic thioredoxin	−240	[52]
Grx1	*E. coli*	Cytosolic glutaredoxin	−230	[53]
AtAPR1C	*A. thaliana*	Chloroplastic glutaredoxin	−188	This study
Grx3	*E. coli*	Cytosolic glutaredoxin	−180	[53]
Grx5	*S. cerevisiae*	Mitochondrial glutaredoxin	−175	[54]

AtAPR1C, AtAPR1 C-terminal redox domain.

## Supplementary Materials

The following are available online at https://www.mdpi.com/2076-3921/8/10/461/s1, Figure S1: Ramachandran plot for crystal structure of AtAPR1 redox domain. Table S1: DALI comparisons using the structure of AtAPR1 redox domain protein that is closest to the mean of the ensemble PDB Z-score sequence identity protein. Table S2: RMSD calculation of four helices between AtAPR1 redox domain protein and the structural relatives.
