# Supplementary material for: C-Terminal Redox Domain of Arabidopsis APR1 Is a Non-Canonical Thioredoxin Domain with Glutaredoxin Function
Source: Antioxidants (Basel). 2019 Oct 8;8(10):461. doi: 10.3390/antiox8100461 (PMC6827007; doi:10.3390/antiox8100461)
Supplement: Supplementary file 1 [file antioxidants-08-00461-s001.pdf]

Supplementary Information

C-terminal redox domain of *Arabidopsis* APR1 is a non-canonical thioredoxin domain with glutaredoxin function

Fang-Fang Chen[1,#], Chia-Yu Chien[1,#], Chao-Cheng Cho[2], Yu-Yung Chang[1], and Chun-Hua Hsu[1,2,*]

[1]Department of Agricultural Chemistry, National Taiwan University, Taipei 10617, Taiwan

[2]Genome and Systems Biology Degree Program, National Taiwan University and Academia Sinica, Taipei 10617, Taiwan

[#]These two authors contribute equally.

[*]Corresponding author:

andyhsu@ntu.edu.tw (C.-H. Hsu).

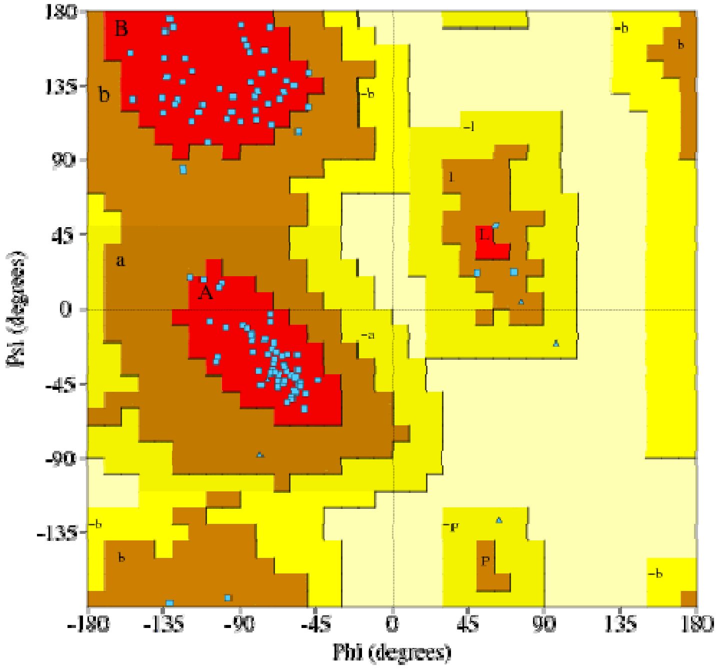

Supplementary Figure S1.

Ramachandran plot for the phi-psi values of the crystal structure of AtAPR1 redox domain. This figure was produced using PROCHECK.

Supplementary Table S1.

DALI comparisons using the structure of AtAPR1 redox domain protein that is closest to the mean of the ensemble PDB Z-score sequence identity protein

Symbol	PDB	Z-score	RMSD	%id	description
A	5YRY			This study	The C-terminal redox domain of APR1 from *Arabidopsis thaliana*
B	3Q6O	13.9	2.3	19%	Sulfhydryl oxidase 1 from *Homo sapiens*
C	4P2L	13.8	2.4	20%	Sulfhydryl oxidase 1 from *Rattus norvegicus*
D	3APQ	13.7	2.4	21%	J-Trx1 fragment of ERdj5 (ER-resident protein disulfide reductase) from *Mus musculus*
E	3APS	13.2	2.6	20%	TRX4 domain of ERdj5 (ER-resident protein disulfide reductase) from *Mus musculus*
F	1EP7	12.9	2.5	15%	H-type Thioredoxin from *Chlamydomonas reinhardtii*
G	4EF0	12.8	2.7	25%	First catalytic domain of protein disulfide isomerase P5 from *Homo sapiens*
H	2YOI	12.7	2.4	14%	LECA (Last Eukaryotes Common Ancestor) Thioredoxin

Abbreviations:

Z-score: normalized score that depends on the size of the structures

RMSD: root-mean-square deviation of Cα atoms in the least-squares superimposition of the structurally equivalent Cα atoms

%id: percentage of identical amino acids over all structurally equivalent residues

Description: the COMPND record from the PDB entry

Supplementary Table S2.

RMSD calculation of four helices between AtAPR1 redox domain protein (PDB code: 5YRY) and the structural relatives.

		RMSD values			
Symbol	PDB	α1 helix (7-17)	α2 helix (33-51)	α3 helix (65-74)	α4 helix (98-111)
A	5YRY				
B	3Q6O	12.421	0.778	6.558	4.507
C	4P2L	6.960	0.948	6.649	4.681
D	3APQ	4.552	0.679	2.819	4.317
E	3APS	11.044	0.920	9.356	3.869
F	1EP7	5.190	1.872	4.615	3.049
G	4EF0	8.774	0.951	9.430	3.970
H	2YOI	13.664	1.838	4.079	4.343

RMSD calculation was performed by using PyMol.
